# Protein–protein interaction network architecture of human polygenic traits reveals domain-spanning connectivity and evolutionary pressures

**DOI:** 10.1186/s13059-026-04253-1

**Published:** 2026-08-27

**Authors:** Ehsan Tamandeh, Kiran Kunwar, Jessica Bigge, Adrian Serohijos, Johannes Schumacher, Carlo Maj, Pouria Dasmeh

**Affiliations:** 1 Center for Human Genetics, Marburg University, Marburg, Germany; 2 Department of Biochemistry, Faculty of Medicine, University of Montreal, Montreal, Canada

## Abstract

**Background:**

Human polygenic phenotypes arise from the combined effects of many genes that interact within molecular networks. Yet, we know little about how the structure of these networks constrains or facilitates the evolution of complex traits. Here, we systematically examine the relationship between protein–protein interaction (PPI) network architecture and evolutionary signatures across 4,756 human polygenic phenotypes.

**Results:**

We show that genes associated with polygenic phenotypes exhibit significantly higher connectivity within the global PPI network compared to matched random gene sets. Highly connected genes are enriched for immune-related biological processes, whereas genes with fewer interactions are preferentially associated with neurogenesis-related functions. Importantly, among trait-associated genes, greater network connectivity is associated with weaker selective constraint, indicating that evolutionary pressure varies according to network embedding.

**Conclusions:**

Together, these findings provide a systems-level framework linking molecular interaction architecture to the evolution of human polygenic traits. To support this effort, we also develop an online portal enabling researchers to generate and explore hypotheses by identifying genes that are both highly associated and highly connected across thousands of polygenic phenotypes.

**Supplementary Information:**

The online version contains supplementary material available at https://doi.org/10.1186/s13059-026-04253-1.

## Background

Most human traits and diseases are polygenic, with multiple genes contributing to their manifestation through regulatory networks that mediate protein–protein interactions [1–6]. Understanding these networks is crucial for gaining mechanistic insights into polygenic phenotypes and for examining how genes associated with these traits may evolve differently from non-associated genes [4, 7, 8]. This is particularly relevant for polygenic phenotypes, where the complex genotype-to-phenotype relationship and the distributed nature of selective signals across many genes make analyses focused on individual genes less informative.

Protein–protein interaction (PPI) networks are broadly categorized into physical and functional interaction networks [9]. Physical interaction networks built from protein–protein interactions map direct molecular contacts between proteins, while functional networks connect genes based on co-expression, shared functions, or regulatory relationships. Both types of networks exhibit modular and hub-like structures, reflecting a common structural pattern in biological networks that likely enhances robustness against perturbations such as mutations and genetic aberrations [10–12]. Although protein–protein interactions have been characterized in several polygenic phenotypes [2, 13–17], we know little about whether and how the number of PPIs formed by genes associated with polygenic traits differs from those of genes not implicated by trait-associated genetic variation. Furthermore, it remains unclear whether genes embedded within these networks are under unique selective constraint depending on their connectivity, potentially influencing their evolution.

The evolutionary constraint acting on a gene is influenced by its position and role within molecular interaction networks, particularly by the number and nature of its interactions with other genes and proteins. Genes occupying highly connected or “hub” positions are subject to a stronger selective constraint, as perturbations in their sequence or regulation can have widespread downstream effects. Consistent with this expectation, empirical studies have shown that genes with many molecular interactions tend to evolve more slowly and are more likely to be essential, reflecting their broader functional impact [18–20]. However, genes also contribute to different traits, each subject to distinct selective pressures. Recent population-genetic studies using genome-wide selection maps have revealed that the strength and direction of selection vary markedly across biological functions, developmental stages, and trait contexts [7, 21–23]. What remains a fundamental, yet underexplored, question is whether—and how—genes harboring trait-associated genetic variants occupy distinct positions within molecular interaction networks, particularly with respect to the relationship between network connectivity and selective constraint.

To know more about PPI networks underlying human polygenic phenotypes, we systematically investigated the structure of these networks across 4,756 traits from the GWAS ATLAS database [24]. We examined the connectivity of highly associated genes within the STRING protein–protein interaction network and contrasted it with that expected under appropriate null models. We find that genes associated with polygenic phenotypes that occupy more highly connected positions in the interactome tend to exhibit weaker selective constraint than less connected genes. Notably, this pattern is specific to associated genes and persists after accounting for the fraction of genes within the MHC locus, as well as for balancing-selection signal and other gene-level covariates. These observations suggest that the relationship between network connectivity and the weaker selective constraint in genes associated with polygenic phenotypes reflects a network-mediated evolutionary signature.

## Results

### Compilation of gene sets and protein–protein interaction networks

**Fig. 1:**
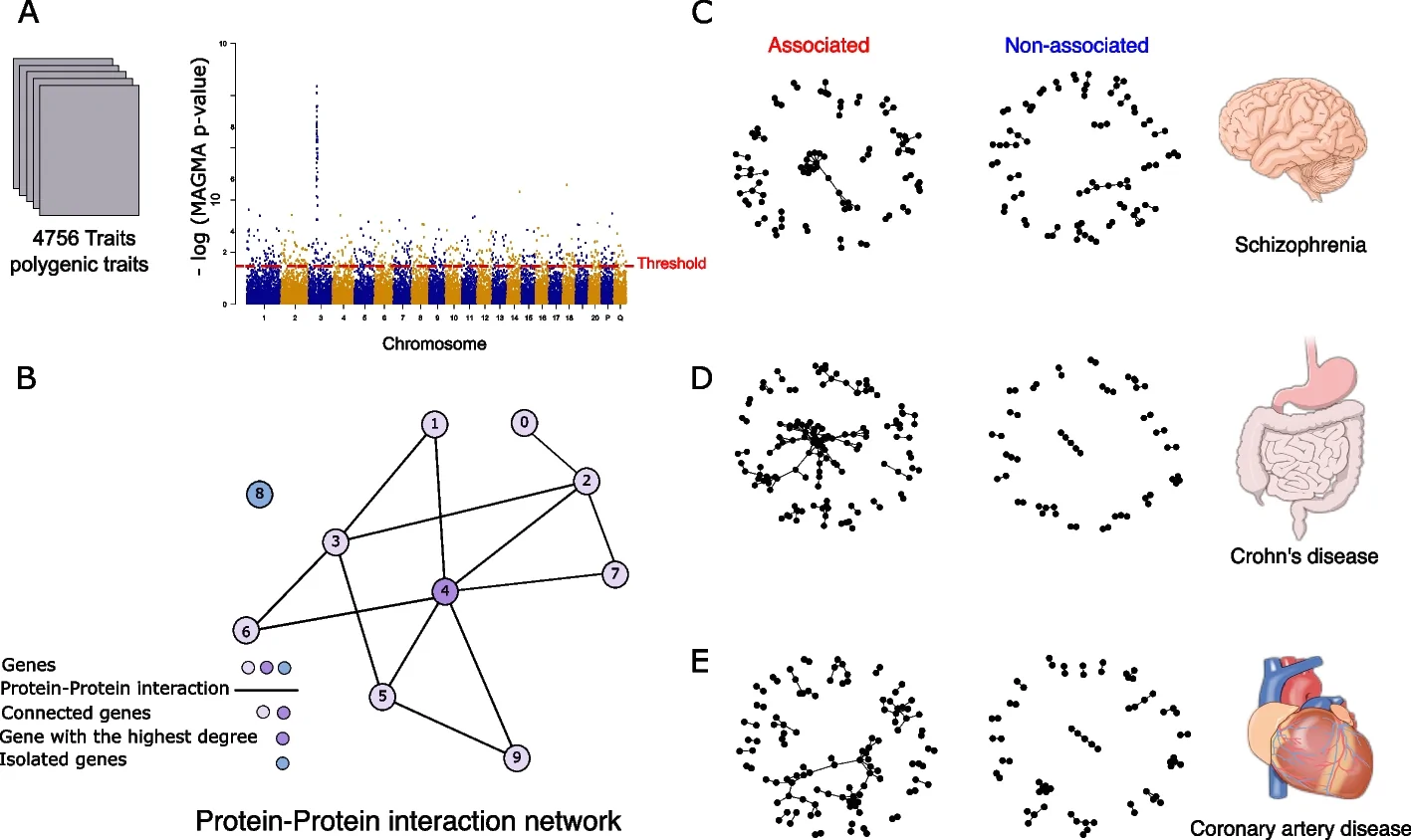
Protein–protein network analysis of human polygenic phenotypes. **A** An overview of our analysis. We identified significant gene–trait associations across 4,756 traits from the GWAS ATLAS and examined their protein–protein interactions. To ensure robust network construction, we focused on traits with at least 60 significantly associated genes. **B** Schematic representation of a protein–protein interaction network, highlighting connected genes, isolated genes, and genes with the highest connectivity. **C**-**E** Protein–protein interaction networks for associated and non-associated genes to schizophrenia (**C**, trait ID 9), Crohn's disease (**D**, trait ID 68), and coronary artery disease (**E**, trait ID 108). These examples illustrate systematic differences in network structure and genetic architecture between associated and non-associated gene sets across representative polygenic diseases. For each trait, associated genes correspond to the top 500 genes ranked by MAGMA significance, while non-associated genes were selected from genes with the highest MAGMA *p*-values. Organ illustrations were obtained from NIAID NIH BioArt library. (Source: bioart.niaid.nih.gov/bioart/228,/212, and/60)

Our aim is to investigate how interaction patterns differ among genes associated with polygenic phenotypes using Protein–Protein Interaction (PPI) networks. To do so, we selected the available human polygenic traits in the GWAS ATLAS (4,756 traits, version database release 3, v2019; Fig. 1A) [24]. GWAS ATLAS compiles this data by calculating the association of human genes (18,476 genes) with each of these traits using MAGMA, a statistical approach that aggregates the effects of SNPs and their association to the trait of interest for each gene [25]. MAGMA assigns a *p*-value to every gene which represents its association with the trait of interest. We chose the genome-wide significance threshold of 2.7 × 10^–6^ (Bonferroni correction for the number of tested genes within MAGMA framework) and selected traits for who at least 60 associated genes were present. This resulted in 461 traits (Additional file 2: Tables S1, S2). We applied this filtering step to reduce the impact of false-positive associations. However, when relevant, we also extended our analysis to the full set of 4,756 polygenic traits by relaxing this constraint and selecting the top 500 associated genes. We chose this fixed number of genes for comparability across traits in our phenome-wide analyses, as this ensures uniformity in the statistical evaluation of network properties across diverse phenotypes. Selecting approximately 500–1000 genes is a common practice in polygenic analyses [26, 27], as it balances statistical power with the ability to capture robust biologically meaningful signals.

We then retrieved interaction scores for these genes from the STRING database [26] and examined their protein–protein interaction networks (see Methods for details) (Fig. 1B). STRING provides both physical protein–protein interactions and functional associations (e.g., co-expression and pathway co-membership); throughout this work, we explicitly distinguish between these interaction types where relevant.

To assess the robustness of our findings to the choice of null model, we compared the connectivity of PPI networks of the 500 most strongly associated genes for each trait to those from 1,000 randomly sampled gene sets of the same size. To control for potential confounding effects of node degree, we additionally implemented degree-preserving randomization, ensuring that the observed differences were not attributable to baseline differences in degree distributions (see Methods).

### Genomic coverage and gene overlap across trait-associated networks

We first assessed the overlap among the top 500 genes used to examine PPI networks across the full set of 4,756 polygenic phenotypes. Of the 18,476 genes catalogued in the GWAS Atlas, 15,904 genes (~ 86%) appear at least once among the PPI networks of top 500 associated genes with these traits. This indicates that, when considering the union of trait-associated networks across phenotypes, a large fraction of the genome contributes to at least one polygenic signal.

We further calculated, for each gene, the number of trait-associated PPI networks in which it appears. The median and mean number of networks per gene are 4 and 11.75 ± 21.71, respectively, indicating that while some genes are associated with many traits, the typical gene participates in only a limited number of trait-associated networks. Notably, even the most recurrent gene appears in at most 303 of the 4,756 networks (~ 6%; Additional file 2: Table S3). Consistent with this observation, pairwise comparisons of the top 500 trait-associated genes revealed modest overlap between traits, with a median of 41 shared genes and a mean of 51.20 ± 41.46 (Additional file 2: Table S4).

Together, these results indicate that trait-associated networks are composed of a wide range of genes and that overlap among traits is broadly distributed rather than concentrated in a small subset.

### Genes associated with polygenic phenotypes exhibit higher protein–protein interaction connectivity than non-associated genes

Using PPI networks of the genes associated with human polygenic phenotypes, we addressed the first question of our study: do genes associated with a given polygenic trait exhibit a higher number of protein–protein interactions compared to non-associated genes? To address this question at the level of single traits, we focused on three representative polygenic diseases drawn from distinct biological domains: coronary artery disease, schizophrenia, and Crohn’s disease (Fig. 1C–E). These traits exhibit low pairwise genetic correlations (*R*_g_ ≈ 0.03), making them suitable for comparative analysis because their network properties are less likely driven by shared genetic architecture or overlapping gene sets [7, 27]. Consistent with this, overlap among the top 500 associated genes was modest: no pair of traits shared more than 46 genes, and only a single gene was common to all three traits (Additional file 2: Table S5).

For each of these polygenic diseases, we examined PPI networks from two gene sets of equal size: (i) associated genes, defined as the top 500 genes with the smallest MAGMA *p*-values for that disease, and (ii) non-associated genes, defined as the bottom 500 genes with the largest MAGMA p-values (see Methods). Across all three traits, associated genes displayed a higher number of protein–protein interactions (PPIs) and were connected to a greater number of nodes within the interactome compared to non-associated genes (Table 1). For example, in schizophrenia, the network of associated genes contained 86 interactions, compared with 57 interactions among non-associated genes. Similar patterns were observed for coronary artery disease and Crohn’s disease, where associated gene networks contained 151 and 100 interactions, respectively, compared to 34 and 50 interactions among their corresponding non-associated gene networks.

**Table 1:** Characteristics of protein–protein interaction networks for polygenic phenotypes. This table provides a comparative analysis of protein–protein interaction networks associated with schizophrenia (Trait ID 9), Crohn's disease (Trait ID 68), and coronary artery disease (Trait ID 108). For each trait, we list the network type (associated or non-associated), the number of connected and isolated genes, the total count of protein–protein interactions, the highest degree of connectivity among genes, and the slope of the degree distribution on a log–log plot

Traits	Network type	Connected genes (no.)	Isolated genes (no.)	Protein–Protein interactions (no.)	Highest degree	Slope of degree distribution (log–log scales)
Schizophrenia (Trait ID 9)	Associated	94	369	86	11	−1.74
	Non-associated	81	376	57	4	−2.77
Crohn's Disease (Trait ID 68)	Associated	139	315	151	13	−1.79
	Non-associated	57	392	34	3	−3.38
Coronary Artery Disease (Trait ID 108)	Associated	115	355	100	8	−1.97
	Non-associated	65	388	50	5	−2.29

To assess whether these differences reflect changes in overall network topology rather than connectivity alone, we compared the degree distributions of associated and non-associated PPI networks. Both networks followed scale-free degree distributions, a hallmark of biological interaction networks (likelihood ratio test, *p* < 0.05; Additional file 1: Fig. S1). This indicates that the primary distinction between the PPI networks of associated and non-associated genes lies in the number of interactions, rather than in a fundamental difference in network structure.

### Phenome-wide enrichment of protein–protein interactions among trait-associated genes

We next extended our analyses from three representative polygenic phenotypes to a phenome-wide scale. To this end, we examined protein–protein interaction networks from the top 500 highly associated genes for each of the 4,756 traits included in the GWAS Atlas, spanning diverse biological and clinical domains (Additional file 1: Fig. S2). In parallel, and where indicated, we analyzed a restricted subset of 461 traits for which at least 60 genome-wide significant associated genes were available, thereby focusing on well-powered GWASs and reducing the influence of sparse or weak association signals. To assess whether the observed network connectivity of trait-associated genes exceeds that expected by chance, we adopted randomly sampled gene sets as our primary null model. Specifically, for each trait we compared the connectivity of networks built from the 500 most strongly associated genes to those constructed from 1,000 randomly sampled gene sets of equal size drawn from the genome (see Methods).

**Fig. 2:**
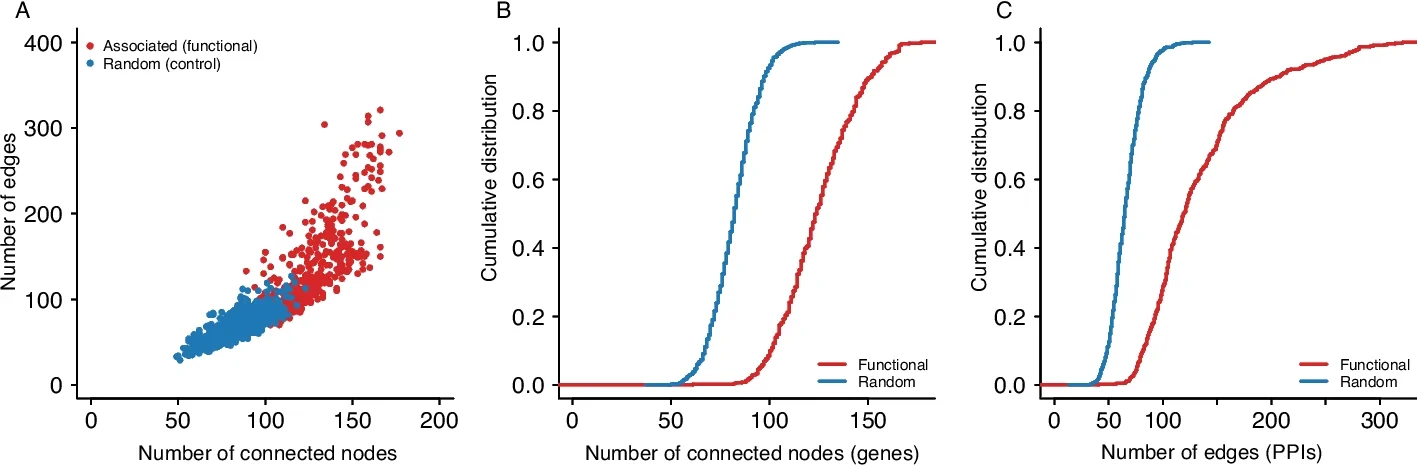
Comparison of protein–protein interaction network features for associated genes and PPI networks of random genes. **A** The number of protein–protein interactions versus the number of connected genes in the interaction networks of associated genes (shown in red) and non-associated genes (shown in blue) with human polygenic phenotypes. **B**,** C** Cumulative distribution function (CDF) plots comparing the distribution of the number of connected nodes (panel B), and the number of edges (panel C) in the protein–protein interaction networks of associated genes with polygenic phenotypes (in red), with random PPI networks (in blue). For both associated and random gene sets, networks were constructed using 500 genes and interaction scores derived from the STRING functional association dataset. Network properties of associated genes differ markedly from those of non-associated and random gene sets, with all comparisons showing strong statistical significance (Wilcoxon rank-sum test, *p* < 10⁻^16^)

We observed that the PPI networks of trait-associated genes exhibited a significantly higher number of interactions and a greater number of connected nodes than expected under the random-gene null model (Wilcoxon rank-sum test, *p* < 10–16; Fig. 2, Additional file 1: Fig. S3, Additional file 2: Table S6). This enrichment was consistent across different domains of complex traits (Additional file 1: Fig. S4, Additional file 2: Table S7), indicating that increased network connectivity is a robust and general feature of genes associated with polygenic phenotypes.

A potential concern is that random gene sampling does not preserve the underlying degree distribution of the PPI networks. To address this, we applied a second approach based on a degree-preserving randomization procedure (see Methods). Specifically, for each trait, we examined the combined network including 500 associated genes and 500 randomly sampled genes and then tested whether the within-network modularity of the associated-gene module exceeded that expected by chance. As a null model, we generated degree-preserving randomized networks using the Maslov–Sneppen rewiring algorithm, in which pairs of edges are repeatedly swapped while prohibiting self-loops and duplicate edges, thereby maintaining the exact degree of every node (see Methods for details). These analyses revealed that the modularity of trait-associated genes was significantly higher than expected under degree-preserving randomization (*p* < 10^–16^, Wilcoxon rank-sum test), corresponding to a significant modularity enrichment in 94.8% of traits (Additional file 1: Fig. S5).

A second potential concern is that increased connectivity could be driven by redundancy among genetically similar traits, such that shared genetic architecture leads to repeated inclusion of the same or closely related genes across traits. To explicitly control for this possibility, we implemented a data-driven strategy based on genome-wide genetic correlations of traits in the GWAS Atlas [24]. We performed hierarchical clustering of GWAS phenotypes based on pairwise genome-wide genetic correlations, thereby grouping traits into clusters of genetically similar phenotypes (Additional file 1: Fig. S6). To ensure that densely sampled or highly correlated phenotype clusters did not disproportionately influence our results, we adopted a conservative subsampling strategy in which a single trait was randomly selected from each genetic-correlation cluster. To assess robustness to the choice of clustering resolution, this procedure was repeated across multiple cut thresholds corresponding to genetic correlation levels of *r*_g_ > 0.10, 0.25, 0.40, 0.55, 0.70, and 0.85. Across all clustering resolutions, PPI networks of genes associated with the subsampled polygenic traits consistently exhibited a significantly higher connectivity and the number of interactions compared to the random PPI networks (Additional file 1: Fig. S7).

Altogether, these results demonstrate that the higher number of PPIs and modular structure of trait-associated genes are robust to alternative null models and reflect a non-random feature of the PPI networks underlying complex human phenotypes.

### Robustness of PPI network connectivity patterns to study- and tissue-specific biases

To ensure that the observed differences in the number of interactions in the PPI networks of the associated genes compared to random gene sets are not driven by ascertainment or study bias, we performed a series of complementary analyses addressing potential sources of systematic bias in the underlying interaction data.

Because STRING integrates evidence from multiple channels, including text mining and co-expression, we first restricted the network to physical interactions only. These high-confidence edges represent direct, experimentally supported protein–protein interactions and are not influenced by literature frequency and text mining. We found trait-associated genes to have more protein–protein interactions than randomly selected gene sets (Wilcoxon *p* = 5.17 × 10^–191^, for connected nodes; *p* = 2.52 × 10^–136^ for edges; Additional file 1: Fig. S8), indicating that the enrichment of connectivity among associated genes is not driven by annotation bias.

We next asked whether the same signal is detectable using structure-based interaction data. We mapped associated and random networks onto protein interaction data from AlphaFold compiled in the Predictomes resource [28] (Additional file 1: Supplementary Note 1, Additional file 2: Table S9). This database integrates AlphaFold-Multimer–derived structural models with experimentally validated protein complexes to generate a unified, structure-informed view of protein–protein interactions. Each predicted interaction in Predictomes is scored using the SPOC (Structural Probability of Complex) classifier, which quantifies the likelihood that two proteins form a stable complex. Associated networks showed consistently higher SPOC classifier probabilities (median = 0.452 vs 0.293; paired Wilcoxon *p* = 0.006, FDR-adjusted) as well as stronger AlphaFold confidence metrics (mean ipTM = 0.43 vs 0.39; (paired Wilcoxon *p* = 0.022, FDR-adjusted). Importantly, this pattern persisted when using AlphaFold-only features excluding literature priors (median = 0.131 vs 0.033; paired Wilcoxon *p* = 0.011, FDR-adjusted). These findings demonstrate that the increased connectivity of associated networks is supported by independent structural evidence rather than by an ascertainment bias.

Finally, we tested whether the observed connectivity patterns might be biased by differences in tissue specificity across traits. In principle, traits whose associated genes are restricted to a single tissue could appear to form more densely connected networks simply because tissue-specific proteins often co-participate in shared molecular pathways or protein complexes. Such an effect could generate an apparent increase in the number of interactions without reflecting a true difference in biological integration. To evaluate this possibility, we quantified the degree of tissue specificity for each of the 4,756 traits by identifying the top 500 MAGMA-ranked genes and assessing their enrichment across 35 GTEx v8 tissues using the TissueEnrich package [7, 29]. We then compared the number of PPIs between traits with low and high tissue specificity using multiple thresholds (Additional file 1: Fig. S9). Contrary to the expectation that narrowly expressed, tissue-specific traits might artifactually appear more connected, we observed the opposite pattern: traits enriched across multiple tissues displayed a higher number of interactions. This finding supports the interpretation that connectivity reflects the cross-tissue and pleiotropic influence of hub genes rather than an artifact of tissue-restricted expression.

Together, these analyses demonstrate that the greater connectivity of trait-associated PPI networks persists when controlling for potential biases in literature curation, data ascertainment, and tissue specificity.

### Selective constraint on genes associated with polygenic phenotypes varies with protein–protein interaction network connectivity

We then turned to the second question of our study: whether genes associated with polygenic phenotypes are subject to differential selection pressures that stem from their placement within PPI networks? This question arises because, traditionally, a relationship has been observed between network connectivity and evolutionary rate, whereby genes with higher connectivity are under stronger selective constraint and tend to evolve more slowly [18, 30]. However, our previous work and that of others suggests that genes associated with polygenic phenotypes may be subject to distinct selective pressure compared to non-associated genes with such traits or genes linked to monogenic diseases [7, 21, 23, 31, 32]. For instance, we have shown that the degree of association between human genes and polygenic phenotypes exhibits significant heterogeneity in its correlation with evolutionary rate [7]. We therefore set out to investigate whether association with polygenic phenotypes modifies the canonical relationship between gene connectivity and selective constraint.

Our initial goal was to replicate the well-established observation that genes with a higher number of interactions tend to evolve under stronger selective constraint compared to genes with fewer interactions, independent of any trait association. To this end, we calculated the average interaction count (average degree in the network) for 15,062 human genes associated with polygenic traits across our subset of 461 phenotypes supported with well-powered genome-wide association studies. This approach is analogous to a mean-field approximation in statistical physics, in which the effects of individual interactions are averaged, allowing the general influence of connectivity on selective constraint to be assessed. We then correlated these average interaction counts with the probability of loss-of-function intolerance (pLI), a widely accepted measure of selective constraint in human genes [33]. The pLI metric estimates a gene’s intolerance to loss-of-function (LoF) mutations, such as protein-truncating variants, frameshifts, and essential splice-site mutations. A pLI score near 1 indicates a high intolerance to LoF mutations, while a score closer to 0 suggests tolerance, implying that such variants are either neutral or only weakly deleterious. Our findings confirmed that genes with a higher average interaction count tend to have high pLI values (*R* = 0.17, *p* < 10^–16^, Spearman’s rank-sum test; Additional file 2: Table S10), supporting the canonical view that genes with more interactions are under stronger selective constraint. This relationship is independent of trait association, as it reflects an average across all traits.

**Fig. 3:**
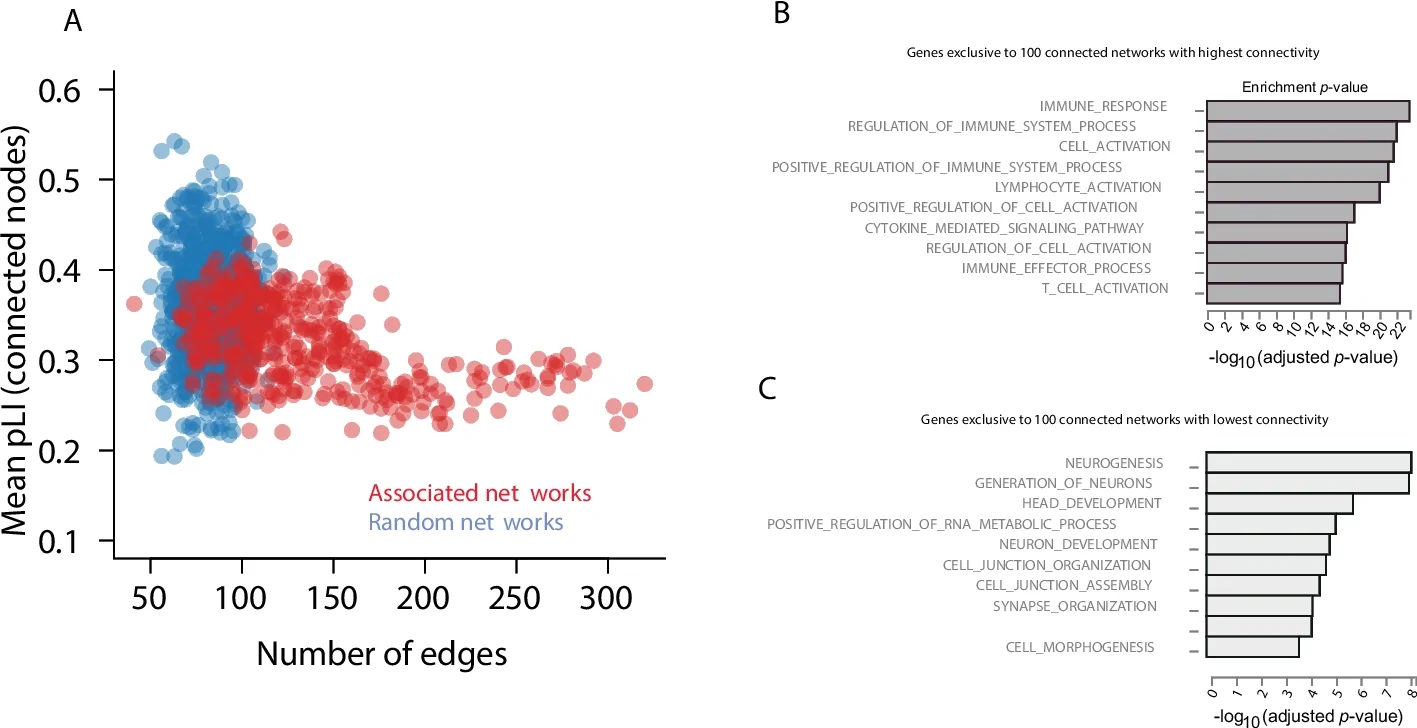
Selective constraint on genes associated with human polygenic phenotypes. **A** The mean probability of loss-of-function intolerance (pLI) of genes within associated (in red) and random (in blue) protein–protein interaction networks plotted against the number of interactions (edges) in these networks. The Spearman’s correlation between the mean pLI and the number of edges in these networks are *R* = −0.44 (*p* = 4.32 × 10^–34^), and *R* = −0.0039 (*p* = 0.90), for the associated and random PPI networks, respectively. **B**,** C** The enrichment of gene ontology (GO) terms related to biological processes within genes exclusive to 100 connected networks with the highest connectivity (panel B, *n* = 870 genes), and 100 networks with the lowest connectivity (panel C, *n* = 3100 genes). In both panels B and C, the x-axis shows -log_10_ of the adjusted *p*-value calculated from a hypergeometric test using Functional Mapping and Annotation of Genome-Wide Association Studies (FUMA)

We then examined how our results might change when accounting for the association of human genes with polygenic phenotypes. Specifically, we tested whether PPIs are linked to differences in the selective constraint of genes associated with polygenic traits. To this end, we calculated the correlation between the average pLI of genes within PPI networks and the number of interactions in each network. Here, deviating from mean-field behavior, we observed that genes associated with polygenic traits forming PPI networks with a higher number of interactions displayed, on average, a lower average pLI (*R* = −0.44, *p* = 4.32 × 10^–34^, Spearman’s rank correlation; Fig. 3A, Additional file 2: Table S11). This inverse relationship was specific to genes associated with polygenic phenotypes and was not observed in PPI networks of randomly selected genes (*R* = −0.0039, *p* = 0.90, Spearman’s rank correlation).

We further repeated this analysis with another measure of selective constraint but focused on protein-coding sequences. We calculated the correlation between the number of interactions and the normalized ratio of the rate of nonsynonymous to synonymous mutations, d*N*/d*S* [34–37]. This ratio, d*N*/d*S*, is commonly used to assess the impact of natural selection on protein-coding sequences: values of d*N*/d*S* < 1 indicate purifying selection, while values of d*N*/d*S* = 1 or > 1 indicate neutral evolution or positive selection, respectively [7, 36–42]. Consistent with our results based on pLI, we observed that genes embedded in highly connected PPI networks associated with polygenic phenotypes tend to have a higher average d*N*/d*S* (*R* = 0.37, *p* = 7.93 × 10^–17^, Spearman’s rank correlation; Additional file 1: Fig. S10). In contrast, no such relationship was observed in random networks (*R* = 0.017, *p* = 0.54, Spearman’s rank correlation).

To better understand why genes in PPI networks associated with polygenic traits exhibit reduced selective constraint at higher levels of interaction, we next examined whether their functional characteristics differ from those of genes in more weakly connected networks.

### Influence of immune genes and balancing selection on network-level selective constraint

To uncover the biological basis underlying the relationship between the number of protein–protein interactions (PPIs) and selective constraint, we examined whether specific functional classes of genes drive this pattern. We therefore focused on two groups of traits: those with the highest and those with the lowest number of PPIs among their associated gene sets (100 traits each). We then identified genes uniquely present in high-PPI versus low-PPI networks to determine whether distinct biological processes account for their differences in network organization. Genes exclusive to PPI networks with a higher connectivity were significantly enriched for immune-related processes, including immune response, immune system process, and immune cell activation, particularly lymphocyte and T cell activation (Fig. 3B). These gene sets prominently contained *HLA* genes and key mediators of immune signaling such as *IL* and *STAT* family members, forming local network clusters corresponding to the JAK–STAT (*p* = 9.0 × 10⁻⁹) and interleukin (*p* = 3.3 × 10⁻⁷) pathways. In contrast, genes exclusive to lowly connected networks were enriched for neurodevelopmental processes, including neurogenesis, neuron differentiation, and synapse organization (Fig. 3C). Repeating the analysis using the average degree of each gene across all networks yielded consistent results. Specifically, when selecting the 500 genes with the highest and lowest average degree, genes with the highest mean of degree distribution across traits were predominantly expressed in immune cell types, whereas those with the lowest mean degree showed preferential expression in neuronal cells (Additional file 1: Fig. S11).

The strong overrepresentation of immune-related genes among highly connected networks suggests that the reduced selective constraint observed for these genes reflects the distinct evolutionary dynamics of immune loci, particularly genes within the major histocompatibility complex (MHC). These genes are known to evolve under long-term balancing selection that maintains allelic diversity within populations [43]. Such evolutionary forces can produce patterns that resemble reduced selective constraint when assessed using metrics such as pLI or d*N*/d*S*, which primarily capture purifying selection [44, 45].

To disentangle these effects, we first removed all genes within the extended MHC region (chr6:25–34 Mb) from each trait network and calculated the change in pLI (ΔpLI) and the change in the number of edges (ΔEdges). This exclusion increased the mean pLI and decreased the number of edges, with the changes in these measures inversely correlated (ΔpLI vs. ΔEdges: Spearman’s *R* = –0.54, *p* < 10^–16^; Additional file 1: Fig. S12). These findings indicate that MHC genes contribute substantially to the observed pattern of lower constraint in highly connected networks. In a multiple linear regression model accounting for network size (nodes^2^), the fraction of MHC genes was the strongest predictor of the number of edges (*β*_MHC_ = 123 ± 3.55, *p* < 2 × 10^–16^). Nevertheless, pLI also remained significantly and negatively correlated with the number of edges (*β*_pLI_ = –17 ± 2.85, *p* = 3.77 × 10⁻⁹), indicating that networks with higher connectivity continue to exhibit reduced selective constraint even after adjusting for the fraction of MHC genes.

It is possible, however, that signals of balancing selection are not confined to the MHC region and that a broader genomic measure could capture additional effects. To directly assess the contribution of balancing selection beyond MHC content, we applied BetaScan2, a population-genetic statistic that detects genomic regions under long-term balancing selection based on polymorphism data [46]. By calculating the average of BetaScan2 scores across genes within each PPI network, we tested whether the weaker constraint observed in highly connected PPI networks could be explained by an enrichment of balancing-selection signals using multiple linear regression. We further incorporated additional covariates into the regression, including average gene expression (from GTEx v8) [47], gene length, and gene evolutionary age (from Phylostrata homology groups) [48], alongside mean pLI, mean BetaScan2 score (B2), network size (nodes^2^), and MHC genes, thereby constructing a comprehensive model to disentangle the relative contributions of these factors.

In this model (edges ~ pLI + B2 + nodes^2^ + mhc + age + exp + length), all predictors together explained a substantial proportion of the variance in the number of edges (adjusted *R*^2^ = 0.83, *p* < 2.2 × 10^–16^). Among biological predictors, MHC content and pLI had the largest effect magnitudes (*β*_MHC_ = 114.3 ± 3.31, t = 34.5, *p* < 2 × 10^–16^; *β*_pLI_ = –12.44 ± 2.10, t = –5.93, *p* = 3.1 × 10^–9^). Gene expression (*β* = 2.67 ± 0.46, t = 5.86, *p* = 4.8 × 10⁻⁹) and gene age (*β* = 2.22 ± 0.64, t = 3.46, *p* = 5.4 × 10⁻^4^) also showed significant positive effects, indicating that highly expressed and evolutionarily older genes tend to participate in more interactions. In contrast, gene length exhibited a significant negative association with connectivity (*β* = –1.86 × 10⁻^5^ ± 5.31 × 10⁻⁶, t = –3.50, *p* = 4.8 × 10⁻^4^). The coefficient for BetaScan2 (*β* = 0.70 ± 0.37, t = 1.86, *p* = 0.06) was marginally positive, suggesting a modest but non-significant contribution of loci under balancing selection. Together, these results indicate that although MHC composition, BetaScan2 signal, expression, gene age, and gene length contribute to connectivity, selective constraint (as measured by pLI) is consistently lower in highly connected networks and this association remains robust after adjustment.

## Discussion

Our findings reveal that protein–protein interaction networks derived from genes associated with polygenic traits exhibit a distinctive organization, characterized by a significantly higher number of interactions than expected under appropriate null models based on randomly selected gene sets matched for size. This enrichment is observed consistently across a wide range of traits and biological domains and remains robust after accounting for genetic similarity among phenotypes.

We also observed a negative correlation between network connectivity and selective constraint among genes associated with polygenic traits. Specifically, associated genes with polygenic traits with more protein–protein interactions tend to have lower pLI values, indicating greater tolerance to loss-of-function variation. Importantly, this pattern persists after accounting for MHC content, signals of balancing selection, and gene-level confounders such as expression level, gene length, and evolutionary age. This reduction in apparent selective constraint could reflect the influence of network architecture on the fitness consequences of gene perturbations. In densely interconnected systems, functional effects may be distributed across multiple interacting components, potentially reducing the marginal fitness impact of variation in any single gene. Theoretical and empirical work on biological robustness has shown that network organization can buffer deleterious perturbations through compensatory interactions, redundancy, or modular structure [49–52].

Balancing selection represents another evolutionary process that can influence patterns of apparent selective constraint, particularly given the prominent contribution of genes from the MHC locus within highly connected networks. Although our analyses did not find that balancing selection fully accounts for this effect, more sensitive scans could attribute a larger fraction of this signature to balancing selection. Current estimates suggest that long-term balancing selection leaves detectable footprints in only a small fraction of human protein-coding genes, roughly 1–8% of the total [43]. However, if balancing selection is also prevalent in non-coding regions of the genome [53]—where the majority of GWAS signals reside— it may explain a larger portion of the observed association between network connectivity and reduced pLI. This could, in principle, reflect the influence of balancing selection acting on regulatory variation. Future studies integrating locus-specific balancing-selection statistics with fine-mapped regulatory annotations will be essential to disentangle these effects.

In this work, we focused on tissue specificity as a potential source of bias that could inflate the observed enrichment of protein–protein interactions among genes associated with polygenic phenotypes and showed that our results are robust to this bias. At the same time, tissue and cell-type specificity represents a fundamental layer in the biological interpretation of polygenic traits. It is well established that GWAS signals are enriched in specific tissues and cell types [54–57], reflecting the cellular contexts in which genetic perturbations are most likely to influence disease risk [58–60]. Accordingly, a natural extension of our findings is to examine how signatures of selection among disease associated genes vary across tissues and cell types. Stratifying variant-level measures of selection—including estimates of purifying or stabilizing selection derived from models such as the α model, which quantifies how the strength of selection scales with allelic effect size [21, 61]— by tissue- and cell-type–resolved regulatory or expression annotations provides a framework for assessing how evolutionary pressures differ across disease-relevant tissues and cell types.

**Fig. 4:**
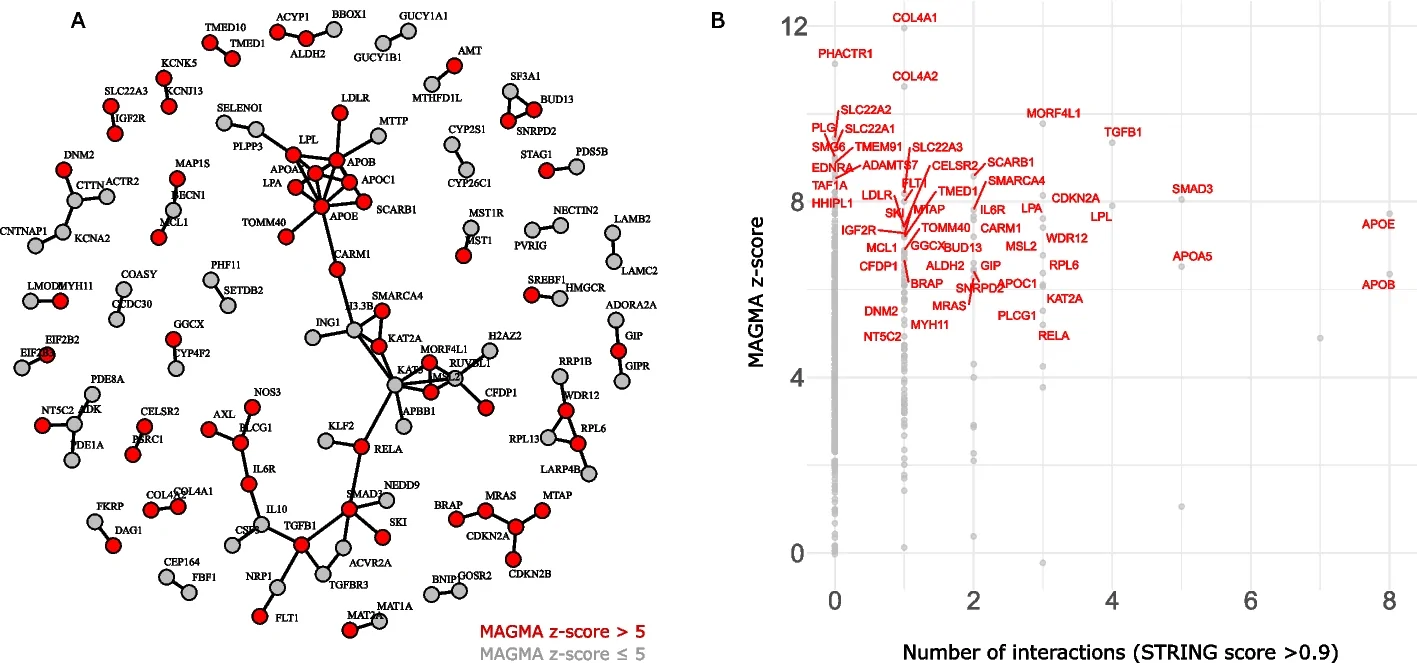
Protein–protein interaction networks facilitate the prioritization of GWAS-identified genes. **A** The interaction network of genes associated with coronary artery disease (Trait ID 108 in the GWAS Atlas). Genes with a MAGMA z-score > 5 are highlighted in red. **B** Scatter plot of MAGMA z-scores against the number of protein–protein interactions for coronary artery disease-associated genes. Genes with a MAGMA z-score > 5 are labeled in red. This figure demonstrates that the number of interactions varies among highly associated genes, providing a framework for gene prioritization in fine-mapping analyses. For example, APOE (encoding Apolipoprotein E), SMAD3 (encoding a key signal transducer for transforming growth factor beta receptors), and LPL (encoding lipoprotein lipase) are highly associated genes with the largest number of interactions in this network

Protein–protein interaction networks further enable us to move beyond linear genomic annotations. Genetic variants often act through epistatic interactions or influence phenotypes via shared biological pathways—relationships that traditional fine mapping approaches may overlook while network-based analyses account for these complex interaction patterns. To illustrate this concept, we plotted the protein–protein interaction network for coronary artery disease (CAD) (Fig. 4). Notably, connectivity within this network was highly heterogeneous; a subset of hub genes, including APOE (encoding Apolipoprotein E), SMAD3 (encoding a key signal transducer for transforming growth factor beta receptors), and LPL (encoding lipoprotein lipase) are highly associated genes with the largest number of interactions in this network. These hub genes are well-established players in lipid metabolism and inflammation—key pathways in CAD pathogenesis [62, 63]. This example demonstrates that prioritizing genes based on their network connectivity and degree of interaction, particularly in highly connected nodes, could help prioritize genes with disproportionate influence on phenotypic variation and trait heritability.

Lastly and to facilitate hypothesis generation in this field, we developed NetPolyGen (accessible at http://netpolygen.com), a publicly accessible web portal that presents the network structure and properties of protein–protein interaction networks associated with human polygenic traits. By integrating network-based insights, NetPolyGen enables researchers to identify highly connected, trait-associated genes and prioritize those most likely to be functionally significant. This resource aims to advance the study of complex trait biology by providing a systems-level framework for understanding genetic architecture and guiding future experimental and evolutionary investigations.

## Conclusions

Our study reveals a general relationship between protein–protein interaction network architecture and the evolutionary properties of genes associated with human polygenic phenotypes. Across thousands of traits, trait-associated genes form more highly connected interaction networks than expected by chance, a pattern that remains robust across alternative null models and after accounting for potential ascertainment and tissue-specific biases. Moreover, among trait-associated networks, greater connectivity is associated with weaker selective constraint, contrasting with the canonical relationship observed across genes genome-wide. This association persists after accounting for MHC content, balancing-selection signals, gene expression, gene age, and gene length. Together, these findings suggest that the evolutionary constraints acting on genes underlying polygenic traits depend not only on individual gene properties but also on their broader molecular network context. NetPolyGen provides a resource for exploring these relationships across human complex traits and for prioritizing trait-associated genes based on both genetic association and network connectivity.

## Methods

### Compilation of data and construction of interaction networks

To analyze the structure and topology of protein–protein interaction networks for human traits, we used the GWAS ATLAS database (version database release 3, v2019), which includes 4,756 polygenic traits and associations for 18,476 genes using MAGMA [25] *p*-value for human gene in relation to the trait of interest [24, 45]. To ensure robust associations, we applied a genome-wide significance threshold of 2.7 × 10^–6^ (Bonferroni correction for the number of tested genes within MAGMA framework) and focused on traits with at least 60 associated genes, reducing our analysis to 461 traits (see Additional file 2: Table S1). For each of these traits, we examined the number of protein–protein interactions using data from the STRING database (version 12.0, 2023), combining physical and functional interaction scores with high confidence (STRING combined score > 0.9). To control for potential biases arising from text mining, co-expression patterns, or pathway-based functional annotations, we further restricted our analyses to the STRING physical interaction subnetwork, which is primarily supported by experimental and curated database evidence of direct molecular binding.

We examined the degree distribution of each network by fitting three types of distributions: a power-law distribution, which corresponds to the degree distribution of scale-free networks; a Gaussian distribution, which represents the random network structure of the Erdos–Renyi model; and a log-normal distribution, often observed in biological networks with heavy-tailed distributions, such as metabolic networks. To assess the best-fitting model, we compared the likelihood of each fit using the Akaike Information Criterion (AIC).

We applied the gene-analysis module from the MAGMA tool to identify gene-association for coronary artery disease (CAD) [25]. MAGMA computes *p*-values per gene by aggregating SNP-level association statistics within or near each gene to create a gene-level test statistic. An empirical null distribution is generated through permutations or simulations to represent the expected distribution of the test statistic under the null hypothesis. The *p*-value for each gene is then calculated as the proportion of null distribution values that are equal to or higher than the observed test statistic, indicating the gene's statistical significance in association with the trait or disease of interest. In order to account for correlations between nearby genetic variants, MAGMA incorporates linkage disequilibrium structure of the regions. In our analysis, we accounted for linkage disequilibrium structure using the European LD matrix from Phase 3 of 1000 Genomes as provide by MAGMA [25]. We used the genome-wide association study (GWAS) summary statistics from the Cardiogram consortium (available in: https://cardiogramplusc4d.org) [64].

### Measures of selective constraint

For the correlation between evolutionary rate and the number of protein–protein interactions, we used the dataset of Chakraborty et al. [65]. In this dataset, the average ratio of nonsynonymous substitutions per nonsynonymous site (d*N*) to synonymous substitutions per synonymous site (d*S*) is used as a metric for assessing the evolutionary rate of distinct genes. To ensure robustness, genes with a minimum of three orthologous pairs across the following species were considered: Macaque (Macaca mulatta), Gorilla (Gorilla gorilla), Orangutan (Pongo abelii), Chimpanzee (Pan troglodytes), and Gibbon (Nomascus leucogenys). The d*N*/d*S* values are then computed using the PAML suite [66], and the average pairwise values of d*N*/d*S* for each human gene (total of 15,248) is calculated. To quantify selective constraint on genes at the level of human populations, we used the probability of loss-of-function intolerance (pLI) metric derived from the Exome Aggregation Consortium (ExAC) dataset [67]. The pLI score estimates the probability that a gene is intolerant to heterozygous loss-of-function (LoF) variants under a population genetic model comparing observed versus expected numbers of protein-truncating variants. Genes with pLI ≥ 0.9 are typically classified as LoF-intolerant, consistent with strong purifying selection against disruptive mutations.

### Network modularity analysis

To test whether trait-associated genes exhibit a stronger modularity than expected by chance, we first constructed, for each trait, a combined network that included the set of 500 associated genes and a random set of 500 genes sampled across the human genome. We then asked whether the modular separation between these two sets is larger than expected under random connectivity while preserving the degree distribution of the network using the Maslov–Sneppen degree-preserving rewiring algorithm [68]. In this procedure, pairs of edges are repeatedly selected, and the edges are swapped provided that no self-loops or duplicate edges are introduced which preserves the exact degree of every node. Each null network was generated by performing $10\times {n}_{edge}$ edge-swap attempts (where ${n}_{edge}$ is the number of edges with the rewiring multiplier = 10). This level of randomization is widely used in network analyses to ensure sufficient mixing while maintaining computational efficiency. For each randomized network, we computed the modularity of the associated-gene module, ${M}_{i}$, defined as:

1 $$M=\frac{1}{2m}\sum\nolimits_{i,j}\left({A}_{ij}-\frac{{k}_{i}{k}_{j}}{2m}\right)\hspace{0.17em}\delta \left({c}_{i},{c}_{j}\right)$$

where ${A}_{ij}$ denotes the presence (or weight) of an edge between nodes i and j; ${k}_{i}$ and ${k}_{j}$ are the degrees (or weighted degrees) of nodes i and j; m is the total number of edges in the network; and $\delta ({c}_{i},{c}_{j})$ equals 1 if nodes i and j belong to the same community and 0 otherwise. We then calculated an empirical z-score (and the corresponding *p*-value) for each trait as

2 $$z=\frac{{M}_{obs}-{\mu}_{null}}{{\sigma}_{null}}$$

where ${M}_{\mathrm{obs}}$ is the observed modularity, and ${\mu}_{\mathrm{null}}$ and ${\sigma}_{\mathrm{null}}$ denote the mean and standard deviation of modularity across all null networks.

### Gene ontology and functional enrichment analysis

We used Functional Mapping and Annotation of Genome-Wide Association Studies (FUMA) [69] to calculate Gene Ontology (GO) enrichment for genes associated with different GO terms, selecting key biological processes as the most insightful terms. Protein-coding genes served as the background set, and gene expression data from 30 general tissue types in the GTEx database (version 8) were used for the expression analysis. To account for multiple testing, we applied the Benjamini–Hochberg false discovery rate (FDR) correction, setting a maximum adjusted *p*-value threshold of 0.05 and requiring a minimum overlap of 2 genes with the gene sets.

### Statistical analysis and network adjacency matrices

All analyses were conducted in R (v4.3.3). The scripts to query the STRING database and construct networks are available in our GitHub repository: https://github.com/EhsanTamandeh/PolyGenNet [70]. The adjacency matrices for the interaction networks are available for download at our webserver: http://netpolygen.com

## Supplementary Information


Additional file 1: Supplementary Information, including Figures S1–S12, Supplementary Note 1, and References.Additional file 2: Supplementary Tables, including Tables S1–S11. 

## Data Availability

The NetPolyGen source code is publicly available on GitHub at https://github.com/EhsanTamandeh/NetPolyGen [70] and is distributed under the MIT License. The exact version of the source code used in this study has been archived on Zenodo and is permanently available at https://doi.org/10.5281/zenodo.21529454 [71]. The adjacency matrices generated during this study have been deposited in Zenodo and are available at https://doi.org/10.5281/zenodo.21530317 [72]. The GWAS summary statistics for 4576 polygenic traits analyzed in this work were taken from the GWAS Atlas (https://atlas.ctglab.nl) [24]. Any additional data generated during this study are available from the corresponding author upon request.
