# Supplementary material for: Protein–protein interaction network architecture of human polygenic traits reveals domain-spanning connectivity and evolutionary pressures
Source: Genome Biol. 2026 Aug 27;27:279. doi: 10.1186/s13059-026-04253-1 (PMC13523357; doi:10.1186/s13059-026-04253-1)
Supplement: Supplementary file 1 [file 13059_2026_4253_MOESM1_ESM.docx]

*Supplementary information*

**Protein-Protein Interaction Network Architecture of Human Polygenic Traits Reveals Domain-Spanning Connectivity and Evolutionary Pressures**

Ehsan Tamandeh^1^, Kiran Kunwar^1^, Jessica Bigge^1^, Adrian Serohijos^2^, Johannes Schumacher^1^, Carlo Maj^1^, Pouria Dasmeh^1^

^1^Center for Human Genetics, Philipps University of Marburg, Marburg, Germany. ^2^Department of Biochemistry, Faculty of Medicine, University of Montreal, Montreal, Canada.

**Figure S1**. **Degree distributions of associated and non-associated protein-protein interaction networks follow power-law distributions.** Degree distribution of associated (in red) and non-associated (in blue) protein-protein interaction networks in Schizophrenia (panels A, B), coronary artery disease (panels C, D), and Crohn’s Disease (panels E, F). The *x*-axis represents the degree of each node, while the *y*-axis shows the number of nodes with the corresponding degree. The degree distribution of both types of networks in all three diseases follows a power-law distributions. The presence of these distributions suggests a scale-free structure in both network types. Both network types are built using the high confidence functional interaction data (combined score > 0.9) from the STRING database^1^.

**Figure S2**. **Representative protein-protein interaction networks of associated genes with polygenic traits.** The figure shows representative protein-protein interaction (PPI) networks, illustrating the diversity in connectivity patterns. These networks exhibit a higher number of nodes and edges, compared to the PPI networks built from random gene sets (Figure 2 in the main text) an increased presence of hub nodes, which play a central role in network connectivity. The presence of these hubs suggests a scale-free structure in the PPI networks. The full set of associated PPI networks are accessible in the web portal <http://netpolygen.com>. We used the package igraph (v0.11.6, 2025; <https://igraph.org>) for network analysis and visualizations.

**Figure S3. Cumulative distribution of network connectivity in associated and random PPI networks using functional interaction scores.** Empirical cumulative distribution functions (ECDFs) of the number of connected nodes (top) and the number of edges (bottom) in trait-associated (red) and random (blue) PPI networks. Each curve represents the cumulative fraction of traits below a given connectivity value. PPI networks of associated genes exhibit systematically higher connectivity, reflected by a right-shift in both distributions (Wilcoxon *p* = 3.03 × 10^-48^ for the number of connected nodes; *p* = 5.91 × 10^-16^ for the number of edges), indicating that trait-associated gene networks are more densely interconnected. Networks were constructed using genes associated with 461 well-powered GWAS traits, with random networks generated using matched gene sets to control for network size.

**Figure S4.** **The connectivity of PPI networks across trait domains.** For each trait domain, we show the mean number of connected nodes (*x*-axis) and the mean number of edges (*y*-axis) in protein–protein interaction (PPI) networks of trait-associated genes (blue) and from matched random gene sets (gray). Each point represents the average across networks within a domain, with error bars indicating variability across traits. Only domains represented by at least 10 traits within the set of 461 well-powered GWASs were included. Across all domains, trait-associated networks exhibit higher connectivity than random networks, reflecting increased interaction density among genes linked to polygenic phenotypes. Domain-specific Wilcoxon *p*-values are shown in Table S7. Networks were constructed using genes associated with 461 well-powered GWAS traits, with random networks generated using matched gene sets to control for network size.

**Figure S5. Modularity of protein-protein interaction networks associated with polygenic traits.** A) Comparison between the observed modularity (Q) of trait-associated PPI networks (left) and the mean modularity of degree-preserved null networks (right). Each dot represents a trait PPI network; diamonds indicate mean values. PPI networks of associated genes display markedly higher modularity than expected by chance, indicating a significant community structure in the organization of associated genes. B) Distribution of –log_10_(*p*) values for modularity enrichment across all traits. The dashed line marks the nominal significance threshold (*p* = 0.05). The majority of networks (94.8%) show significantly higher modularity compared to their null expectation, suggesting that polygenic trait-associated genes cluster into densely connected modules rather than being randomly distributed across the interaction space.

**Figure S6.** **Trait clustering based on genetic similarity.** We converted the pairwise genome-wide genetic correlations between GWAS phenotypes into a distance metric ($d_{ij}=1-r_{g}(i,j))$, where $r_{g}(i,j)$ denotes the genetic correlation between traits *i* and *j*. Traits were grouped based on this distance using hierarchical clustering. The horizontal dashed line indicates an example cut threshold corresponding to $r_{g}=0.5$, used to partition traits into clusters of genetically similar phenotypes. To assess robustness to clustering resolution, we repeated the analysis across multiple genetic-correlation thresholds ($r_{g}>0.10, 0.25, 0.40, 0.55, 0.70, and 0.85$). At each threshold, one representative trait was randomly sampled from each cluster, ensuring that no cluster of genetically correlated phenotypes disproportionately influenced the connectivity analyses.

**Figure S7. Robustness of network connectivity enrichment to trait clustering resolution.** Each panel shows the relationship between the mean number of connected nodes (x-axis) and the mean number of edges (y-axis) for networks constructed from genes associated with GWAS traits (blue) and randomly matched control genes (gray), after reducing trait redundancy using genetic-correlation–based hierarchical clustering. Panels correspond to different genetic correlation cut thresholds (*r*_g_ > 0.10, 0.25, 0.40, 0.55, 0.70, 0.85), where the threshold determines the height at which the dendrogram (Figure S6) is cut. Importantly, lower *r*_g_ thresholds correspond to higher cuts in the dendrogram, resulting in fewer clusters, as multiple correlated traits are grouped together. For each threshold, one representative trait is sampled per cluster, such that the number of points reflects the number of non-redundant trait clusters rather than the number of individual traits exceeding a given *r*_g_ value. Highlighted points with error bars indicate the mean connectivity across subsampled trait sets (blue) and random networks (gray), with error bars denoting ±1 standard deviation. Across all clustering resolutions, genes associated with subsampled, non-redundant trait sets consistently occupy more highly connected regions of the interaction network compared to random controls. with fdr adjusted p-values of 6.12×10^-15^, 3.10×10^-28^, 4.10×10^-42^, 1.75×10^-48^, 1.04×10^-57^, 4.32×10^-75^ for traits samples with different genetic correlation cut thresholds of *r*_g_ > 0.10, 0.25, 0.40, 0.55, 0.70, 0.85, respectively.

**Figure S8. Cumulative distribution of network connectivity in associated and random PPI networks using physical interaction scores.** Empirical cumulative distribution functions (ECDFs) of the number of connected nodes (top) and edges (bottom) in trait-associated (red) and random (blue) PPI networks. Each curve represents the cumulative fraction of traits below a given connectivity value. Associated networks exhibit systematically higher connectivity, reflected by a right-shift in both distributions (Wilcoxon *p* = 5.17 × 10^-191^ for connected nodes; *p* =2.52 × 10^-136^ for edges), indicating that trait-associated gene networks are more densely interconnected.

**Figure S9. Relationship between tissue specificity and network connectivity across GWAS traits.** Empirical cumulative distribution functions (ECDFs) showing the distribution of the number of connected nodes for traits categorized by tissue specificity at three thresholds (A–C). “Low” and “High” groups correspond to traits with fewer or greater than the indicated number of enriched tissues, respectively. Across all thresholds, traits that show gene enrichment across many tissues (high group, shown in red) tend to have more protein-protein interactions.

**Figure S10**. The average ratio of non-synonymous to synonymous substitution rates (d*N*/d*S*) for genes within associated (in red) and random (in blue) PPI networks plotted against the number of interactions (edges) in these networks. The Spearman’s correlation between the mean dN/dS and the number of edges in these networks are *R* = 0.37 (*p*=7.93×10^-17^), and *R* =0.017 (*p*=0.54), for the associated and random PPI networks, respectively.

**Figure S11. Tissue enrichment of genes within protein-protein interaction (PPI) networks.** A) Bubble plot showing the enrichment of the top 500 genes with the highest average PPI degree across GTEx tissues. The color scale represents the false discovery rate (FDR), and bubble size indicates the number of genes contributing to each enrichment term. B) Bubble plot showing the enrichment of the 500 genes with the lowest average degree across GTEx tissues. The color scale indicates FDR, and bubble size reflects the number of genes in each tissue.

**Figure S12. Effect of removing MHC genes on network connectivity and selective constraint.** Scatterplot showing the relationship between the change in mean selective constraint (ΔMean pLI) and the change in network connectivity (ΔEdges) after removing all genes from the extended MHC region (chr6:25–34 Mb) across 4,576 trait networks.

**Supplementary Note 1**

To test whether the higher number of interactions observed for the associated networks was also reflected in structure-supported protein–protein interactions, we mapped both associated and non-associated networks to the Predictomes/SPOC resource^2^, which links approximately 40,000 AlphaFold-Multimer–predicted complexes to experimentally supported protein–protein interactions (PPIs). This approach provides a SPOC (Structure-Predicted Orthogonal Confidence) score, representing the probability (0–1) that a predicted complex corresponds to a true physical interaction based on structural and evolutionary features. After filtering out traits without matched gene pairs in either network type, 290 traits remained for analysis. Across these, associated networks consistently showed higher SPOC classifier scores than their non-associated counterparts (Table S9). The median combined SPOC probability was 0.452 vs. 0.293 (paired Wilcoxon *p*= 0.006, FDR-adjusted), and even when restricted to AlphaFold-Multimer–only features—excluding literature and omics priors—associated networks retained significantly higher values (0.131 vs. 0.033, *p* = 0.011, FDR-adjusted). Associated networks also exhibited higher AlphaFold confidence metrics (mean ipTM = 0.43 vs. 0.39) and greater model convergence (mean avg_n_models = 0.40 vs. 0.33). Together, these findings indicate that trait-associated gene networks are enriched for structurally supported interactions, and that this enrichment persists even when using purely structure-based metrics, suggesting it is not attributable to literature ascertainment bias.

**References**

1	Mering, C. v., Huynen, M., Jaeggi, D., Schmidt, S., Bork, P. & Snel, B. STRING: a database of predicted functional associations between proteins. *Nucleic acids research* **31**, 258-261 (2003).

2	Schmid, E. W. & Walter, J. C. Predictomes, a classifier-curated database of AlphaFold-modeled protein-protein interactions. *Molecular cell* **85**, 1216-1232. e1215 (2025).
